# Measuring directional urban spatial interaction in China: A migration perspective

**DOI:** 10.1371/journal.pone.0171107

**Published:** 2017-01-31

**Authors:** Fangzhou Li, Zhiming Feng, Peng Li, Zhen You

**Affiliations:** 1 Institute of Geographic Sciences and Natural Resources Research, Chinese Academy of Sciences, Beijing, China; 2 University of Chinese Academy of Sciences, Beijing, China; Shanxi University, CHINA

## Abstract

The study of urban spatial interaction is closely linked to that of economic geography, urban planning, regional development, and so on. Currently, this topic is generating a great deal of interest among researchers who are striving to find accurate ways to measure urban spatial interaction. Classical spatial interaction models lack theoretical guidance and require complicated parameter-adjusting processes. The radiation model, however, as proposed by Simini et al. with rigorous formula derivation, can simulate directional urban spatial interaction. We applied the radiation model in China to simulate the directional migration number among 337 nationwide research units, comprising 4 municipalities and 333 prefecture-level cities. We then analyzed the overall situation in Chinese cities, the interaction intensity hierarchy, and the prime urban agglomerations from the perspective of migration. This was done to ascertain China’s urban spatial interaction and regional development from 2000 to 2010 to reveal ground realities.

## Introduction

China’s urbanization rate has seen rapid and steady growth over the past two decades, exceeding 50% since 2011 and reaching 56.1% in 2015. This massive urbanization has led the Chinese government to afford more importance to the planning, establishment, and assessment of urban systems, thereby making research on urban systems pivotal to the field of economic geography, urban planning, regional development, and so on. Urban spatial interaction, such as migration[1], trade[2], industrial cooperation[3], and information exchange[4], unites neighboring cities into urban agglomerations and city clusters. Hence, measuring urban spatial interaction can efficaciously shed light on the urban network structure, urban spatial interaction intensity, core city’s influencing spatial scope, and so on[5,6].

Spatial sense is fundamental in interaction research, since it can uncover not only distribution patterns but also variation tendencies[7,8,9]. In spatial interaction, the interaction intensity drops off when the distance between the interacting subjects increases; this phenomenon is called Distance Decay[10]. Urban spatial interaction can be divided into two schools based on the different influence modes that distance exerts. The first school, represented by Reilly’s gravity model[11] and Harris’s potential model[12], assumes that absolute distance and urban size are critical factors in urban spatial interaction. The second school, represented by Stouffer’s law of intervening opportunities, states that the interaction intensity between cities is directly proportional to the opportunity available in the destination city and inversely proportional to the opportunity within the radius of the distance between the origin city and destination city[13]. In other words, it is the relative distance, rather than the absolute distance, that determines interaction intensity. Studies have also indicated that the latter school has delivered better performance than the former in measuring long-range spatial interaction[14,15]. These classical models can reveal and estimate spatial interaction, which is difficult to measure objectively from diverse perspectives and dimensions. However, judging from the theoretical perspective, these classical models, particularly the gravity model, are empirical models that are subjective generalizations of experience rather than rigorous formula derivations. Moreover, judging from the application perspective, the parameter setting of classical models has a significant influence on simulation accuracy, which, nevertheless, always varies with the situation, thus vastly reducing the generalizability of the models[16]. With scholars’ increasing interest in microstudies, such as ethology and econometrics, more research is now focused on the study of the origin and destination of migration, the directional interaction of the network, the direction of diffusion of contagious diseases, and so on. Meanwhile, accurate O-D (Origin-Destination) data are difficult to come by and the traditional approach fails to shed light on the details of urban spatial interaction.

The radiation model was proposed by Simini et al. with a rigorous formula derivation of the probability of random migration[17]. It can simulate directional migration accurately using merely population distribution information since it proves the self-similarity of migration, signifying that migration probability is independent of the population scale of the origin and migration distance. Simini et al. applied the radiation model to simulate intercity migration, intracity commuting, interstate freight, and telecommunication. They also compared the radiation model’s simulation result with that of the gravity model and statistical data to verify the radiation model’s satisfactory simulation effect[17]. In addition, Masucci et al. contrasted the application effect of the radiation model and the gravity model in England, and came to the conclusion that the radiation model performed better in large-scale migration simulation[18]. Truscott and Ferguson’s application of the radiation model to contagious diseases in England and the US also turned out to be optimistic[19]. In China, the radiation model facilitated Hou et al.’s analysis of the intensity of the floating population among 55 rural towns in one city[20]. In view of the radiation model’s high simulation accuracy, low requirement for input parameters, and the ability to procure directional O-D data, it is considered to have excellent application prospects in the research field of spatial interaction, epidemic spreading, urban growth boundary, resource flow, and so on.

In the context of China’s urban-rural dual structure, the floating population can promote exchange of technology and culture among regions, compensate for the urban labor shortage, and relieve the rural labor surplus, thus having a positive effect on the rational allocation and optimization of production factors. Hence, population migration research is accorded great importance by scholars and government officials. Researchers can evaluate urban competitiveness and the connection degree between cities by studying the intensity, pattern, and destination of migration. Decision makers can formulate infrastructure construction, social security, and health policies based on the number and spatial distribution of the migration population. In fact, under the stringent disclosure policy with regard to socioeconomic data in China, only the numbers with regard to the inter-provincial migration population are publicly accessible, which is far from useful in further research. In conclusion, it is, therefore, of great significance to obtain migration population data between prefecture-level cities or even counties in China.

We applied the radiation model in China to simulate the number of directional migration among 337 nationwide research units comprising all 4 municipalities and 333 prefecture-level cities of China. We then analyzed Chinese cities’ overall situation, interaction intensity, and the development status of prime urban agglomerations from the perspective of migration. We not only tested the radiation model’s applicability in China, the world’s most populous nation, but also ascertained China’s urban spatial interaction and regional development over 10 years to reveal the problems on the ground.

## Data and methods

### Research area

The research scope of this paper was mainland China; Taiwan, Hong Kong, and Macao were not included since their statistical standards of demographic data differ from those of mainland China. To ensure comprehensive coverage of the study area, we established 337 research units, including Beijing, Shanghai, Tianjin, Chongqing, the 4 municipalities, and all 333 prefecture-level cities.

**Fig 1:**
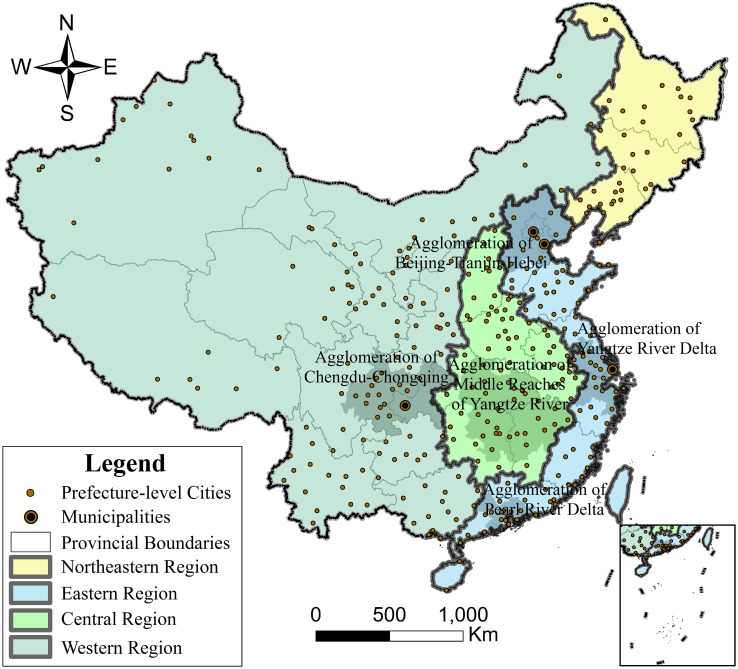
Sketch map of the research area. Prefecture-level Cities, Municipalities, Provincial Boundaries, Northeastern Region, Eastern Region, Central Region, Western Region.

For effective regional management, China is administratively divided into four economic regions–the Eastern Region, Central Region, Western Region, and Northeastern Region–based on social and economic development[21]. Therefore, this zoning method was applied in this paper for regional comparison. Furthermore, the Chinese government, with great effort, has constructed five national urban agglomerations[22]: Agglomeration of Pearl River Delta[23], Agglomeration of Yangtze River Delta[24], Agglomeration of Beijing-Tianjin-Hebei[25], Agglomeration of Chengdu-Chongqing[26], and Agglomeration of Middle Reaches of Yangtze River[27]. These five national urban agglomerations generate approximately half of China’s GDP in a mere one-tenth of the territory, demonstrating their indispensability to China’s development. Therefore, we made a specific analysis of these five national urban agglomerations. An overview of the study area is shown in Fig 1.

We studied and compared the migration intensity in 2000 and 2010 to provide insight into China’s explosive growth over these ten years. According to China’s fifth and sixth national population censuses, from 2000 to 2010, its population increased by 73.9 million at an annual population growth rate of 0.57%, which is nearly half of that of the previous decade. Its floating population, meanwhile, grew by 117 million at an annual growth rate of 6.11%. Thus, it can be seen that in the context of China’s slower-growing population, its population mobility was still markedly increasing. This world’s largest population flow will inevitably exert a profound influence in and beyond China. We tried to analyze the migration between Chinese cities to examine urban competitiveness and connectivity.

### Data description and processing

Demographic data and administrative boundary data were the two types of fundamental data used in this paper. The total population and emigrant population of each research unit in 2000 and 2010 were extracted from China’s fifth and sixth national population censuses, which were conducted in the two respective years (S1 Table). In China, the national population census is the most authoritative and publicly available demographic census.

One noteworthy point is that the definition of emigrants in China’s national population census is people who have lived in places other than the city of their household registration for over six months[28]. China’s population grid data for 2000 and 2010 were provided by the Data Center for Resources and Environmental Sciences of the Chinese Academy of Sciences. The data were interpolated to 1km*1km using the geographic differentiation law based on the nationwide demographic data at a county level. As the sole multi-temporal population grid data on a national scale in China, it subdivided the population census data into grid cells, thus breaking down the barriers of administrative boundaries and providing sophisticated data for population distribution research[29,30].

Meanwhile, the administrative boundary data of China’s prefecture-level cities in 2000 and 2010 were derived from the National Geomatics Center of China[31]. Several prefecture-level cities were renamed, split, or merged between 2000 and 2010; therefore, to maintain consistency in the research result between 2000 and 2010, the administrative boundary data for 2010 were set as the standard and the data for 2000 were processed by changing the rule to fit this standard.

### Methods

#### The radiation model

The radiation model was utilized to simulate the O-D adjacent matrix of migration among the 337 research units. The theory and application process of the radiation model is introduced below. There are two theoretical hypotheses of the radiation model: 1. The emitted particle will be absorbed by the nearest location whose absorption capacity is higher than its threshold. 2. The particle absorption threshold, as well as location absorption capacity, is in direct proportion to its competitive ability. To be specific to demography, the radiation model assumes that migration is a random process dominated by the joint probability of the population distribution of the origin, destination, and involved area. People emigrating from a highly populated city have a higher absorption threshold than those emigrating from a scarcely populated city. Meanwhile, a highly populated city also has a higher absorption capacity than a scarcely populated city. With rigorous derivation based on two theoretical hypotheses, Simini et al. obtained the fundamental equation of the radiation model, shown in Eqs (1) and (2).

$$T_{ij}=T_{i}.P_{ij}$$ (1)

$$P_{ij}=\frac{m_{i}n_{j}}{\left(m_{i}+s_{ij}\right)\left(m_{i}+n_{j}+s_{ij}\right)}$$ (2)

*T*_*ij*_ is the total migration from origin *i* to destination *j*; *T*_*i*_ is origin *i*’s total emigrant population; *P*_*ij*_ is the possibility of emigrating from origin *i* to destination *j*; *m*_*i*_, *n*_*j*_ separately refer to origin *i*’s and destination *j*’s total population; and *s*_*ij*_ is the total population in the circle of radius *r*_*ij*_, which means that the geometric distance between origin *i* and destination *j* is centered at *i* (excluding the origin and destination population)[17].

We established the adjacent matrix using Python, then utilized Eqs (1) and (2) to calculate the number of migrants flowing between every two research units, which included the 4 municipalities and 333 prefecture-level cities. *T*_*i*_, *m*_*i*,_ and *n*_*j*_ were directly available from China’s national population census, while *s*_*ij*_ could not be directly acquired. Therefore, we used China’s population grid data to calculate *s*_*ij*_. First, we set research unit *i* as the center to draw a circle whose radius was the geometric distance between origin *i* and destination *j*. Then, we removed research unit *i* and *j*’s area in the circle to finalize the statistical zone. Finally, we added the total population of the statistical zone in China’s population grid data to obtain *s*_*ij*_.

#### The complex network indicators

By the end of the twentieth century, complex network theory had developed rapidly with the propositions of the BA network[32] and SW network[33]. Since its essence was consistent with social networks, complex network theory was brought into many socio-economic fields such as viral transmission network[34,35,36,37,38], social relation network[39], and international trade network[40]. We built a complex network when analyzing the five national agglomerations, where cities are represented as nodes and the migration population between cities is represented as edges, whose directions denote the population flows and whose weights denote the population size. We used the network’s weighted degree, weighted clustering coefficient, and centralization to estimate the linkage intensity, aggregation degree, and internal coordination among the five national urban agglomerations.

The network’s weighted degree is defined as Eq (3). The weighted degree of a node is the sum of the weight of the edges linked with the node. The network’s weighted degree is the average of its nodes’ weighted degree:

$$k_{w}=\sum_{i=1}^{N}k_{i}/N$$ (3)

where *k*_*w*_ is the network’s weighted degree and *k*_*i*_ is the weighted degree of node *i*. In an urban network study, the network’s weighted degree can reflect cities’ linkage intensity.

The network’s weighted clustering coefficient is defined as Eq (4). The node’s clustering coefficient is the proportion of observed triangles among all possible triangles involving the node. The node’s weighted clustering coefficient considers not only link status but also link strength. The network’s weighted clustering coefficient is the average of its nodes’ weighted degree and it shows the global connection property in the network[41].

$$C_{w}=\frac{\sum_{r\Delta }w}{\sum_{r}w}$$ (4)

where *C*_*w*_ is the network’s weighted clustering coefficient, Σ_*r*Δ_*w* is the total weight of all possible triangles in the network, and Σ_*r*_*w* is the total weight of the linked triangles in the network. In an urban network study, the higher the network’s weighted clustering coefficient, the more prosperous the sub-group in urban agglomerations.

The network centralization is defined as Eqs (5) and (6). It expresses the degree of inequality or variance in the network as a percentage of that of a perfect star network of the same size. The network centralization is 1 for a network with star topology and 0 for a network where each node has the same connectivity. This means that the higher the network centralization, the bigger the difference between the degree of its nodes:

$$Cd_{i}=\frac{k_{i}}{N-1}$$ (5)

$$C=\frac{\sum_{i=1}^{N}Cd_{max}-Cd_{i}}{N-2}$$ (6)

where *C* is the network centralization, *Cd*_*i*_ is the degree of centrality of node *i*, and *Cd*_*max*_ is the largest degree of centrality in the network. In an urban network study, excessively large network centralization demonstrates that core cities are unduly aggregative, while excessively small network centralization indicates core cities’ unobvious regional advantage.

## Results and analysis

The radiation model was applied to simulate the number of migrants flowing between every two units of the 337 research units both in 2000 and in 2010. Based on 226,464 simulation results, we utilized spatial analysis methods and complex network indicators to ascertain China’s urban spatial interaction from the perspective of the overall situation, intensity hierarchy, and prime urban agglomeration.

### Analysis of the overall situation

#### 1. Holistic analysis of the number of immigrants

**Fig 2:**
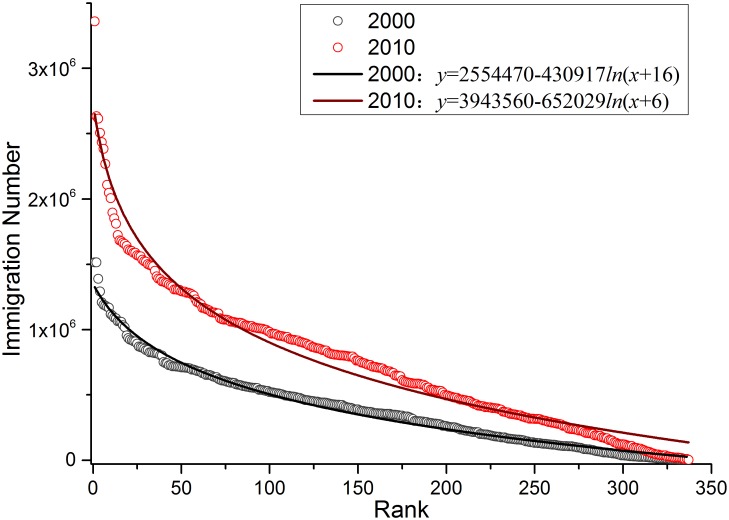
Scatter plot of urban immigration numbers in 2000 and 2010. 2000, 2010, 2000: *y* = 2554470-430917*ln*(x+16), 2010: *y* = 3943560-652029*ln*(x+6).

To analyze China’s urban immigrants, in this section, we summed up the 226,464 O-D results with same destination to obtain the number of immigrants from the 337 research units in 2000 and 2010, and then compared the results of the two years. The results revealed that during the 10 years, migration intensity rose significantly while the Matthew Effect in China’s urban development was aggravated. The average number of urban immigrants was 391,996 in the year 2000 and 736,296 in the year 2010, whose increasing amplitude reached 87.83%. Fig 2, which depicts the fitting logarithm curve of ranked scatter of immigration numbers in 2000 and 2010, shows a much higher urban primacy index in 2010, a larger scale of urban immigrants in 2010 from the bigger Y-intercept, and a wider scale gap of urban immigrants in 2010 from the larger variable amplitude. Therefore, it can be concluded this is the Matthew Effect in China’s urban development, a widening urban competitiveness gap with more exoteric metropolises and more conservative micropolises.

#### 2. Spatial analysis of major migration

**Fig 3:**
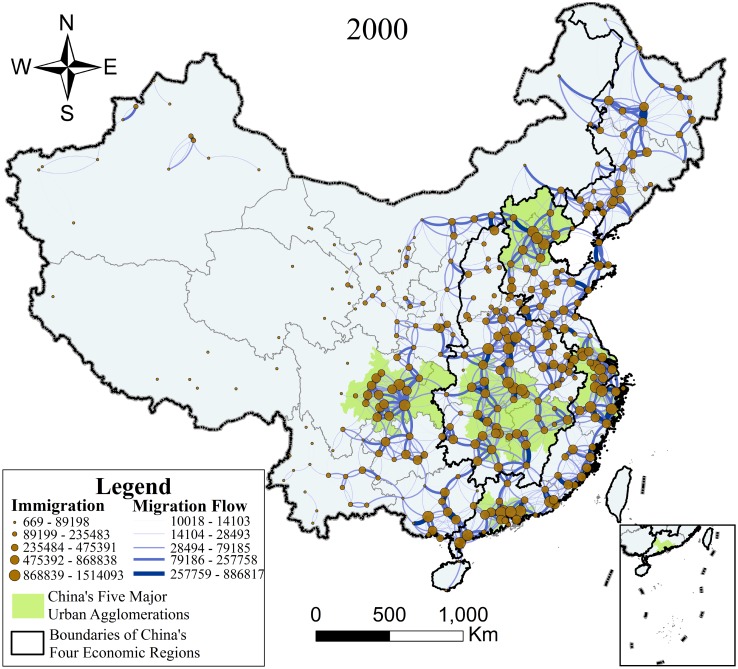
Map of major migration in 2000. Immigration: 669–89198, 89199–235483, 235484–475391, 475392–868838, 868839–1514093, Migration Flow: 10018–14103, 14104–28493, 28494–79185, 79186–257758, 257759–886817, China’s Five Major Urban Agglomerations, Boundaries of China’s Four Economic Regions.

**Fig 4:**
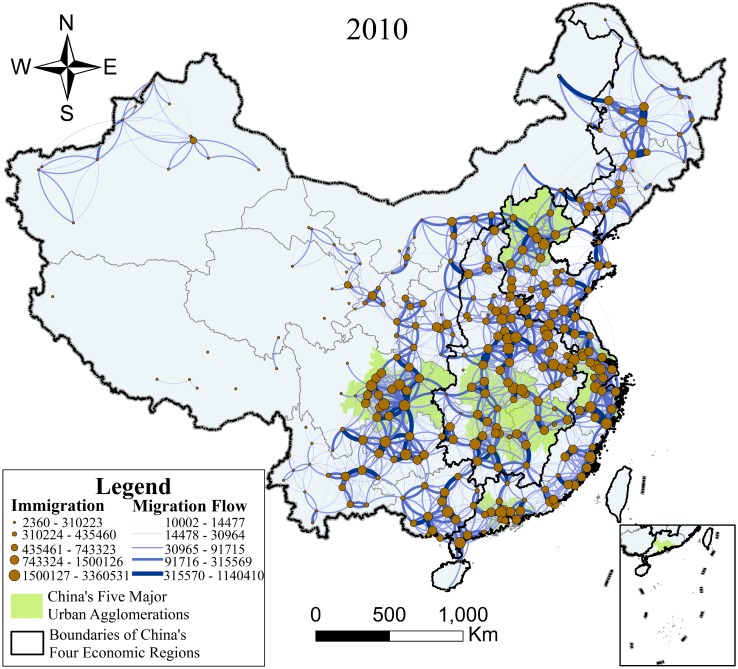
Map of major migration in 2010. Immigration: 2360–310223, 310224–435460, 435461–743323, 743324–1500126, 1500127–3360531, Migration Flow: 10002–14477, 14478–30964, 30965–91715, 91716–315569, 315570–1140410, China’s Five Major Urban Agglomerations, Boundaries of China’s Four Economic Regions.

To eliminate interference from small-scale migration, in this section, we set 10,000 as the lower limit of major migration and obtained 1,805 population flows in 2000 and 2,698 in 2010, as shown in Figs 3 and 4. In Figs 3 and 4, the size of the point represents the urban population scale, the shade and thickness of the curve represents migration intensity, and this is classified into five levels by the Geometrical Interval classification method, which works reasonably well on data that are not distributed normally[42]. In addition, the curve also shows that migration flow occurred in a counterclockwise direction, which means that Figs 3 and 4 not only embodied the urban level but also the intensity and direction of migration.

It can be concluded from Figs 3 and 4 that large-scale migration within China all occurred between the near field, that the migration intensity in the east was much higher than that in the west, and that the population distribution in the east was far denser than that in the west. From the regional perspective, the Eastern Region owned the largest scale of floating population while the Northeastern Region had the smallest, and the five national urban agglomerations showed great capacity for population gathering. On comparing the data across the ten years, it is clear that China’s major migration trends became more frequent and the spatial distribution of migration became more uniform.

As can be seen in Fig 3, in 2000, major migration in China mainly occurred in the Eastern Region and Central Region, especially in the five national urban agglomerations. Of the major migration that took place, 37.45% occurred in the Eastern Region, especially in and around the Agglomeration of Pearl River Delta, Yangtze River Delta, and Beijing-Tianjin-Hebei; 25.99% occurred in the Central Region, especially in and around the Agglomeration of Middle Reaches of Yangtze River; and 24.54% occurred in the Western Region, especially in and around the Agglomeration of Chengdu-Chongqing. Only 12.02% of China’s major migration occurred in the Northeastern Region, and the farther north, the less the migration. This indicates that the five national urban agglomerations possessed great capacity for population gathering, especially the Agglomerations of Pearl River Delta, Yangtze River Delta, Beijing-Tianjin-Hebei, and Chengdu-Chongqing, all of which have both intense internal flow and strong connections with their hinterlands.

From Fig 4, it is clear that, in 2010, as major migration became more frequent and the spatial distribution of migration tended to become more equilibrated, the difference between China’s major migrations in the Eastern, Central, and Western Regions decreased and the migration intensity out of the five national urban agglomerations was clearly on the rise. The average number of major migrations in 2010 was 81,686 and grew by 31.06% from 2000 onward. Of the major migrations took place, 35.40% occurred in the Eastern Region, 2.05 percentage points less than that in 2000. For the Western Region, this was 30.80%, 6.26 percentage points more than that in 2000, where the rapid rise of the Agglomeration of Chengdu-Chongqing exerted significant influence. For the Central Region, this was 24.20%, 1.79 percentage points less than that in 2000. Finally, only 9.60% of China’s major migrations occurred in the Northeastern Region, 2.42 percentage points less than that in 2000, showing that the Northeastern Region was China’s slowest-growing region between 2000 and 2010. Furthermore, there were more dense regions of migration out of the five national urban agglomerations, making the network of China’s major migration more complicated in 2010.

### Analysis of three major intensity hierarchies

**Fig 5:**
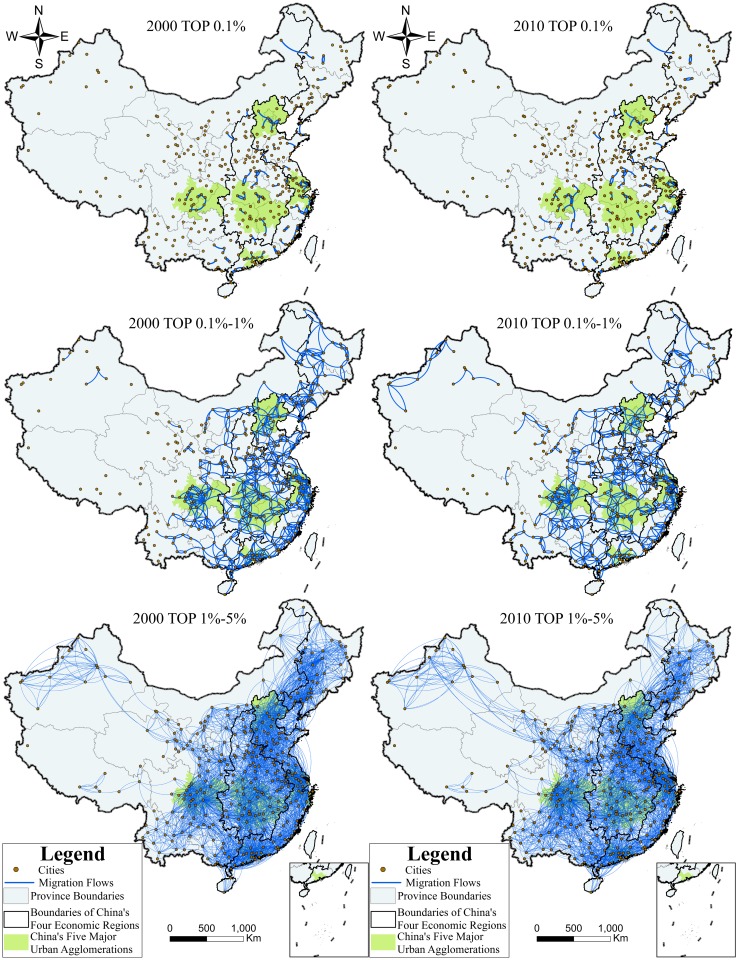
Map of the three intensity hierarchies in 2000 and 2010. Cities, Migration Flows, Province Boundaries, Boundaries of China’s Four Economic Regions, China’s Five Major Urban Agglomerations.

To study the migration of different major intensity hierarchies in China, in this section, we extracted the top 1‰, top 0.1‰-1%, and top 1%-5% that separately represented China’s strongest, second strongest, and moderately strong migrations of the 113,232 results in both 2000 and 2010, as shown in Fig 5. Intensity hierarchy analysis revealed that China’s large-scale migration mainly occurred in the Eastern Region, in or around the five national urban agglomerations, and in the same province or between neighboring provinces. From the perspective of the ten-year comparison, the spatial distribution of China’s large-scale migration became more widespread and equilibrated in 2010. The rise of the Western Region led by the development of the Agglomeration of Chengdu-Chongqing, as well as the universal recession of the Northeastern Region was clearly reflected. Detailed indicators are shown in Table 1.

**Table 1:** Detailed indicators of the three intensity hierarchies in 2000 and 2010.

Hierarchy	1‰		1‰–1%		1%–5%	
Year	2000	2010	2000	2000	2010	2000
**Average migration straight-line distance (km)**	82.31	87.92	129.01	82.31	87.92	129.01
**Eastern Region Migration proportion (%)**	53.98	40.71	36.41	53.98	40.71	36.41
**Central Region Migration proportion (%)**	22.13	23.01	26.89	22.13	23.01	26.89
**Western Region Migration proportion (%)**	16.81	30.97	23.85	16.81	30.97	23.85
**Northeastern Region Migration proportion (%)**	7.08	5.31	12.85	7.08	5.31	12.85
**Five national urban agglomerations Migration proportion (%)**	42.48	33.63	25.81	42.48	33.63	25.81

#### 1. Top 1‰ migration network

The first hierarchy had the shortest migration distance (the average migration straight-line distance was less than 90 km) and China’s most frequent migrations all happened in the near field (migrants all flowed within the same province or between neighboring provinces). This had the most significant spatial concentration where migration intensity in the Eastern Region and the five national urban agglomerations was the highest (53.98% of China’s top 1‰ migration in 2000 occurred in the Eastern Region, 40.71% in 2010; 42.48% of China’s top 1‰ migration in 2000 occurred in the five national urban agglomerations, 33.63% in 2010). The Eastern Region and the five national urban agglomerations had the most suitable residential environment, the most developed economy, and the largest labor markets[43,44]. Therefore, it can be seen that suitability for residence, degree of economic development, and labor market size had a positive correlation with migration scale. From the perspective of the ten-year comparison, the first hierarchy’s migration distribution tended to be more dispersive, wherein the migration intensity in the Western Region was boosted (the Western Region’s share of China’s top 1‰ of migration rose by 14.16 percentage points). On the contrary, the migration intensity in the Eastern Region and the five national urban agglomerations dropped significantly from 2000 to 2010 (the Eastern Region’s share of China’s top 1‰ of migration dropped by 13.27 percentage points, and the five national urban agglomerations dropped by 8.85 percentage points). This reveals that the intensity of concentration of migration distribution in the first hierarchy was reduced.

#### 2. Top 1‰-1% migration network

The migration distance of the second hierarchy was nearly 50% longer than that of the first (the average migration straight-line distance was nearly 130 km), and migrants mostly moved within the same province or between neighboring provinces; a few moved to trans-provinces. The migration distribution of the second hierarchy was the most equilibrated, wherein the migration intensity of the Eastern Region, Central Region, and Western Region was relatively close. From the perspective of the ten-year comparison, driven by the Agglomeration of Chengdu-Chongqing, the Western Region, especially the Southwest Region, enjoyed a remarkable increase in migration intensity (the Western Region’s share of China’s top 1‰-1% migration was boosted by 6.09 percentage points). Conversely, the migration intensity of the Northeastern Region dropped by 3.43 percentage points, indicating that the growth rate of migration intensity in the region was relatively slow. The Chinese government proposed a plan to revitalize the Northeastern Region in 2003. However, seen from the migration perspective, the implementation of the plan was extremely unsatisfactory, which also confirmed the previous research conclusion that the Northeastern Region experienced a cliff-like recession during the ten years[45,46].

#### 3. Top 1%-5% migration network

The migration distance of the third hierarchy was the longest (the average migration straight-line distance was nearly 300 km), and long-distance migration became more common. The migration distribution of the third hierarchy was the most extensive wherein the migration proportion of the five national urban agglomerations was the lowest in the three hierarchies (only less than 14% of China’s top 1%-5% migration occurred in the five national urban agglomerations). It was observed that migration sub-regions, with the five national urban agglomerations at their core, were connected to each other by border cities and played the role of the five national urban agglomerations by becoming the sought-after destinations of migration. From the perspective of the ten-year comparison, there was an increase in long-distance migration in the Northwestern Region and in the short-distance migration in the Southwest Region (the Western Region’s share of China’s top 1%-5% migration increased by 4.07 percentage points), while the share of China’s top 1%-5% migration of the Eastern Region, Central Region, and Northeastern Region all decreased slightly.

### Analysis of the five national urban agglomerations

As mentioned previously, the five national urban agglomerations were the regions in which population flow was the most intense. Therefore, in this section, we focus on the five national urban agglomerations, analyze their effects on migration at the national and regional levels using migration indicators, and ascertain their interconnection and internal structures using three complex network indicators.

**Fig 6:**
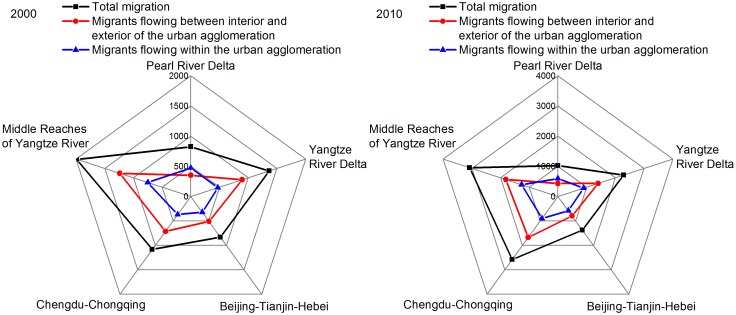
Radar chart of migrant numbers for the five national urban agglomerations in 2000 and 2010. 2000: Total migration, Migrants flowing between the interior and exterior of the urban agglomeration, Migrants flowing within the urban agglomeration, 2010: Total migration, Migrants flowing between the interior and exterior of the urban agglomeration, Migrants flowing within the urban agglomeration.

To carry out research on the five national urban agglomerations, the count of each national urban agglomeration’s immigrations, emigrations, migrants flowing between the interior and exterior of the urban agglomeration, migrants flowing within the urban agglomeration, total number of migrants, and migration intensity in 2000 and 2010 were utilized. The number of migrants flowing between the interior and exterior of the urban agglomerations comprised the sum of the number of immigrants and emigrants, which illustrated its impact on the outside. The number of total migrants was the sum of the number of migrants flowing between the interior and exterior of the urban agglomeration and migrants flowing within the urban agglomeration. The urban agglomeration’s migration intensity comprised the ratio of its total number of migrants to its total population. Moreover, the network’s weighted degree, weighted clustering coefficient, and centralization were utilized. Detailed indicators are as shown in Fig 6, Tables 2–4.

**Table 2:** Detailed indicators of migration numbers for the five national urban agglomerations in 2000.

Indicator	Pearl River Delta	Yangtze River Delta	Beijing- Tianjin- Hebei	Chengdu- Chongqing	Middle Reaches of Yangtze River
**Immigration**	371.95	277.81	133.80	182.05	419.41
**Emigration**	100.34	193.50	189.70	188.53	331.15
**Migrants flowing between interior and exterior of the urban agglomeration**	472.29	471.31	323.50	370.58	750.56
**Migrants flowing within the urban agglomeration**	350.17	894.05	511.50	715.43	1237.76
**Total migration**	822.46	1365.36	835.00	1086.01	1988.32
**Total population**	4287.91	8743.12	7091.29	9959.05	11669.75

**Table 3:** Detailed indicators of migration numbers for the five national urban agglomerations in 2010.

Indicator	Pearl River Delta	Yangtze River Delta	Beijing- Tianjin- Hebei	Chengdu- Chongqing	Middle Reaches of Yangtze River
**Immigration**	478.95	563.17	283.84	470.11	776.81
**Emigration**	116.83	328.94	297.72	435.08	487.56
**Migrants flowing between interior and exterior of the urban agglomeration**	595.78	892.11	581.56	905.19	1264.36
**Migrants flowing within the urban agglomeration**	426.78	1399.96	793.07	1676.14	1815.84
**Total migration**	1022.56	2292.07	1374.63	2581.33	3080.20
**Total population**	5612.73	10763.00	8378.57	9575.89	12110.91

**Table 4:** The three complex network indicators of the five national urban agglomerations in 2000 and 2010.

Indicator		Pearl River Delta	Yangtze River Delta	Beijing- Tianjin- Hebei	Chengdu- Chongqing	Middle Reaches of Yangtze River
**weighted degree**	**2000**	588308	808717.44	1114757.9	625845.63	566737.63
**weighted degree**	**2010**	729998	1224927.1	1143359.6	1452066.6	838177.83
**weighted degree**	**Increasing amplitude**	24.08%	51.47%	2.57%	132.02%	47.90%
**weighted clustering coefficient**	**2000**	18493.15	12729.03	15510.07	13308.68	6986.73
**weighted clustering coefficient**	**2010**	18867.47	15645.55	18550.46	18619.67	8370.54
**weighted clustering coefficient**	**Increasing amplitude**	2.02%	22.91%	19.60%	39.91%	19.81%
**centralization**	**2000**	25.52%	14.05%	35.61%	25.03%	9.10%
**centralization**	**2010**	18.04%	13.46%	36.59%	20.48%	9.09%
**centralization**	**Increasing amplitude**	-29.31%	-4.20%	2.75%	-18.18%	-0.11%

From the perspective of the ten-year comparison, the migration intensity, network’s weighted degree, and weighted clustering coefficients of the five national urban agglomerations evidently increased. The increasing amplitude of the number of migrants flowing between the interior and exterior of the urban agglomeration was pervasively larger than the increasing amplitude of the number of migrants flowing within the urban agglomeration, indicating a remarkable boost in the regional influence and internal connection density of the five national urban agglomerations. The network’s centralization of the five national urban agglomerations, apart from the Agglomeration of Beijing-Tianjin-Hebei, all declined slightly, tending to balance the internal structures of the five national urban agglomerations. Further, the development of the Agglomerations of Yangtze River Delta and the Middle Reaches of Yangtze River was much faster than the development of the Agglomerations of Pearl River Delta and Beijing-Tianjin-Hebei, while the development of the Agglomeration of Chengdu-Chongqing was the fastest, demonstrating its strong development momentum. In addition, the number of migrants flowing in the urban agglomeration of the five national urban agglomerations, except for the Agglomeration of Pearl River Delta, was in every case higher than the number of migrants flowing between the interior and exterior of the urban agglomeration, which means that China’s prime urban agglomeration exerted more of an aggregation effect than a radiation effect.

1. The Agglomeration of Pearl River Delta developed steadily and had a great radiation effect on the region to its exterior. When compared with the other four national urban agglomerations, the Agglomeration of Pearl River Delta’s increasing migration number, whether the number of migrants was flowing between the interior and exterior of the urban agglomeration or within the urban agglomeration, was the smallest, and its increasing amplitude of network’s weighted degree as well as its weighted clustering coefficient were relatively small, which meant that it had entered the stage of steady development. It was the only urban agglomeration in which the number of migrants flowing within the urban agglomeration was lower than the number of migrants flowing between the interior and exterior of the urban agglomeration, which showed that it exerted more of a radiation effect than an aggregation effect. Furthermore, its decreasing amplitude (29.31%), which was the highest, tended to make its internal structure more evenly equilibrated.
2. The Agglomeration of Yangtze River Delta had a high interconnection density and positive development momentum. All migration indicators, network’s weighted degree, and weighted clustering coefficient were universally large, which symbolized a high interconnection density in and around it. The rational network’s centralization (13%-14%) demonstrated a reasonable internal structure. More importantly, despite its vast scale, it also showed the second fastest rate of development.
3. The Agglomeration of Beijing-Tianjin-Hebei showed development into a senior stage; low migration intensity and excessive concentration were the two main problems it had to deal with. Its migration intensity ranked last both in 2000 and 2010, which alerted the government to the need to help promote rational migrant flow. Although its network’s weighted degree and weighted clustering coefficient ranked at the top, their increasing amplitudes were limited, illustrating its senior development stage with slow growth rate. Moreover, the continuing rise of the network’s centralization (the increasing amplitude is 2.75%) reflected that its internal structure tended to show excessive concentration.
4. The Agglomeration of Chengdu-Chongqing developed at breakneck speed and had an equilibrated internal structure and positive development momentum. The increasing amplitude of all migration indicators, network’s weighted degree, and weighted clustering coefficient remained the highest, which revealed its booming development over the decade. Further, the number of immigrants and emigrants was essentially flat, the scale of migrants flowing within the urban agglomeration was tremendous, and the network’s centralization decreased by 18.18%. All these proclaimed its equilibrated internal structure and positive development momentum.
5. The Agglomeration of Middle Reaches of Yangtze River had an enormous population and rapid development, yet its core city lacked sufficient capability for population gathering. The huge scale of its population gave rise to the largest migration indicators of all types. Seen from the complex network indicators, it was in the junior stage of development in 2000, but, nevertheless, thrived during the decade (the increasing amplitude of the network’s weighted degree and weighted clustering coefficient was 47.90% and 19.81%, respectively). Above all, the network’s low degree of centralization (approximately 9%) was proof of its scarce capability for population gathering.

## Conclusion and discussion

### Conclusion

We applied Simini et al.’s radiation model in China to simulate directional migration between 337 nationwide research units comprising all four municipalities and 333 prefecture-level cities to establish the O-D adjacent matrix of migration; we then quantitatively analyzed the overall urban situation, three major intensity hierarchies, and the five national urban agglomerations from the perspective of migration. Finally, we ascertained China’s urban spatial interaction and regional development over 10 years to reveal the on-the-ground realities. The specific conclusions drawn were as follows:

1. In terms of migration distribution, all the large-scale migration in China appeared within the near field, revealing remarkable regional resistance, which had a notable correlation to China’s particular household registration system. Currently, China’s migration distribution shows strong spatial heterogeneity, where the economic development degree has a positive correlation to migration scale. Nationally, the migration intensity in the east was much higher than that in the west and the population distribution in the east was far denser than that in the west. From the regional perspective, the migration intensity in the east was much higher than that in the west and the population distribution in the east was far denser than that in the west.
2. In terms of the ten-year development from 2000 to 2010, China’s migration intensity boosted significantly, and China’s migration distribution tended to become more evenly equilibrated. The Eastern Region was still the most developed with the continuous development of the Agglomerations of Pearl River Delta, Yangtze River Delta, and Beijing-Tianjin-Hebei. The Central Region’s development forged ahead with the lead of the Agglomeration of Middle Reaches of Yangtze River. The Western Region, particularly the Southwestern Region, driven by the Agglomeration of Chengdu-Chongqing, increased rapidly. Last, it remains an undisputed fact that the Northeastern Region was experiencing a general decline.
3. From the perspective of the five national urban agglomerations, China’s best five urban agglomerations possessed a large and growing capacity for population gathering. The Agglomeration of Pearl River Delta developed steadily into the senior stage and had a great radiation effect on its exterior region. The Agglomeration of Yangtze River Delta had a high interconnection density, reasonable internal structure, and positive development momentum. The Agglomeration of Beijing-Tianjin-Hebei did develop into the senior stage; however, low migration intensity and excessive concentration were two main problems in the region. The Agglomeration of Chengdu-Chongqing developed at breakneck speed and it showed an equilibrated internal structure and immense potential. The Agglomeration of Middle Reaches of Yangtze River had an enormous population and rapid development; yet, its core city lacked the capacity for population gathering.

### Discussion

Currently, research on spatial interaction has become more thorough; the symmetrical results obtained using classical models neither matched the reality nor met the need for accurate analysis. As a result, scholars often use specific indicators, such as intercity passenger capacity, relations between headquarters and branch offices, as well as urban connection data based on the newly emerging big data of cyberspace, including check-in data on social networks, GPS trace data, and so on. These indicators can reflect asymmetrical relations and their influence to a certain extent, but conclusions are often one-sided owing to the limitations of the indicators. However, the radiation model can simulate all kinds of asymmetric flow such as population, information, and capital, making it possible to accurately assess the relations between and influences among cities.

For the first time, the radiation model was applied to China, the world’s most populous country with the largest scale of migration. A systematic pectination of the application process was made, providing a paradigm for further application of the radiation model. The results illustrated that the radiation model, which required few model parameters and limited data categories, could function well to show directional and asymmetrical spatial interaction. The radiation model simulated the data of directional migration among China’s prefecture-level cities, which are of great research value in China, where detailed socioeconomic data are difficult to access. Furthermore, we analyzed the simulation migration results in 2000 and 2010 from many aspects, thus drawing sound conclusions from the perspective of regional and urban agglomerations.

Another characteristic of the radiation model is its high simulation efficiency. Satisfactory simulation results can be obtained with a modest amount of known information and no parameter-adjusting process, proving the rationality of the inherent theory of the model. When the prior information is sufficient, the accuracy of the model should further improve by inputting more information. The radiation model determines *s*_*ij*_ by drawing circles, and the premise is the homogeneity of traffic accessibility in the region. As a developing country, China’s huge regional disparity of transport infrastructure will affect the simulation accuracy. Figs 2 and 3 show the frequent migration between the south and north shores of Bohai Bay, especially between Dalian and Weihai, as well as Yantai. The reason for this is that the geographical damping coefficient of seaway transportation is much larger than that of the landway, which is not taken into consideration. Hence, Ren et al. modified the range of *s*_*ij*_ from the same geometric distance to the same commute time scope[47], which was worth attempting and promoting. Furthermore, in order to verify the advantages and disadvantages of the radiation model in a more comprehensive and intuitive way, given the same data and conditions, it is possible to compare the information demand, computational efficiency, and simulation accuracy of the radiation model with those of other spatial interaction models.

It is worth mentioning that China’s frequent migration mostly occurred in the near field; migration distance was short and the migration damping coefficient was large. This migration pattern did not conform to the Random Walking model. Many researchers have also reached a consensus on the fact that China’s strict household registration system has become the biggest obstacle to free migration[48,49]. In recent years, the reform of the household registration system will certainly promote China’s population flow further. In contrast, this phenomenon was inconsistent with the previous research conclusion that the interaction between China’s metropolises was stronger[50,51]. Because Kang and Liu studied urban spatial interaction based on air transportation and Internet communication, their geographical damping coefficient was significantly less than the migration’s. In this paper, the data used were those of the floating population who lived in different cities for more than six months, belonging to the long-term or even permanent population movement, while most of the active population flow between metropolises comprised short-term commuting. Moreover, this process conformed to the Theory of Distance Decay. In urban spatial interaction, there are macro- and microforms of Distance Decay. At the macrolevel, owing to Distance Decay, the interaction intensity between two cities (e.g., migration numbers between two cities) is negatively correlated to distance, under the premise that other variables (e.g., urban population) are relatively stable. At the microlevel, Distance Decay is represented as the probability of individuals migrating to different destinations being negatively correlated to distance.

In terms of research breadth, according to China’s national population census, the floating population is defined as people who live in places other than the cities of their household registration for over six months. Therefore, the simulation floating population scale reflects relatively stable migration, and the final result also indicates China’s large geographical damping coefficient. However, commuter, capital, and information flow is more capable of reflecting industrial and economic cooperation among cities. In future work, different data categories according to research objects can be adopted to create a more comprehensive analysis of spatial interaction in China.

In terms of research depth, we analyzed China’s urban spatial interaction to reveal China’s urban and regional development at the national level. It is obvious that the core urban agglomeration had a huge impact on China’s economic and social development. After understanding the overall situation in China, we will focus on China’s urban agglomerations in future work. Scientific methods will be used to define the spatial boundaries of prime urban agglomerations, a reasonable index system will be put forward to divide the development stage of urban agglomeration, and multiple factors such as natural condition, economic location, and industrial distribution will be combined to analyze their geographical mechanism. All these will provide a persuasive scientific basis and decision support for China’s urban agglomeration planning.

## Supporting information

S1 Table The total population and emigrant population of research unit in 2000 and in 2010. Research unit, 2000: total population, emigrant population, 2010: total population, emigrant population.
(XLSX)
